# Vascularized pedicle iliac bone grafts as a hip-preserving surgery for femur head necrosis: a systematic review

**DOI:** 10.1186/s13018-019-1262-2

**Published:** 2019-08-27

**Authors:** Fan Yang, Qiushi Wei, Xiaojun Chen, Guoju Hong, Zhenqiu Chen, Yaolong Chen, Wei He

**Affiliations:** 1 The First Clinical Medical School, Guangzhou University of Chinese Medicine, Guangzhou, 510405 Guangdong China; 2 Institute of Hip Joint, First Affiliated Hospital of Guangzhou University of Chinese Medicine, Guangzhou, Guangdong China; 3 Key Laboratory of Evidence Based Medicine of Lanzhou University, Lanzhou, Gansu China

**Keywords:** Osteonecrosis, Femoral head, Bone grafts, Vascularized pedicle ilium, Systematic review

## Abstract

**Background:**

Osteonecrosis of the femoral head was gradually concerned as a global disease for its progression to collapse of the femoral head, ultimately causing the arthritic change. Due to the high incidence of this disease in young people, arthroplasty tends to be suspected for its uncertain long-term efficiency. Vascularized pedicle iliac bone grafts, as a hip-preserving surgery, were regarded as an effective option in hip-preserving protocol since the 1970s. Nevertheless, there exist no unified standards widely agreed as the optimal operative program since the lack and heterogeneity of related studies. Thus, we execute this systematic review to synthesize and analyze existing studies, and further suggest a direction of future researches.

**Methods:**

Data were collected by searching electronic database (PubMed, Embase, and Cochrane Library) and including the eligible studies of all types of clinical researches except case report. Through our extraction and synthesis of included study results in respect of clinical evaluation (rating scales), radiographic evaluation, joint survival rate, viability of implanted flap, and complications by transform varied assessment method into a unified standard, we qualitatively analyze and discuss the efficacy of VPIBG according to the quality of individual study and the heterogeneity across the included studies.

**Results:**

Our systematic review includes 1 RCT, 2 case-control studies, and 13 case series studies, resulting in a significant improvement of postoperative scores. Minority of hips progressed for joint replacement. Some researches suggested a high collapse rate in the collapsed femoral head before the operation. Compared with some other hip-preserving surgeries, the complications of VPIBG are relatively slight and barely affect clinical efficiency.

**Conclusions:**

A better clinic response was obtained after this treatment, especially in femoral heads before the appearance of a crescent sign. The fixation of the implanted iliac bone flap increases the clinical effect. The majority of complications were slight and rarely affected clinical efficacy.

**Electronic supplementary material:**

The online version of this article (10.1186/s13018-019-1262-2) contains supplementary material, which is available to authorized users.

## Introduction

Osteonecrosis of the femoral head (ONFH) has been gradually treated as a devastating disease and become an increasing worldwide health problem. Evidences have indicated a nonnegligible morbidity in the USA [1–3], Japan [4], and Korea [5]. The main pathomechanism generally accepted involves a reduction in the blood supply to the femoral head caused by high-dose corticosteroid use [6], alcohol abuse [7], fracture of the femoral head [7, 8], chemotherapy regimens [9], and other unknown etiological that were considered as idiopathic necrosis [10]. For the reason of its pathogenesis is poorly understood and the absence of specific treatment, most cases ultimately progress to femoral head collapse and joint destruction, with hip arthroplasty being the appropriate treatment option [11]. Due to the young age of the patients [4, 5] and uncertainty of long-term survivorship of prosthesis [12], however, concerns regarding the complexity of revision surgery have been gradually highlighted. Thus, there is great interest in procedures, for instance, the joint-preserving surgeries, which could slow disease progression [13].

Vascularized pedicle iliac bone grafting (VPIBG), as a widely used joint-preserving surgery since the 1970s [14], could reduce pressure of the femoral head, diminish intraosseous pressure, provide structural support, and restore vascular supply to enhance lesion healing, therefore enhancing the stability of femoral head structure and preventing collapse or secondary collapse. The deep iliac circumflex artery (DICA), superficial iliac circumflex artery (SICA), and ascending branch of the lateral circumflex artery (ALCA) are generally used as nutrient vessels for the iliac bone flap. Vascularized muscle-pedicle bone flap was also considered to be a kind of vascularized pedicle iliac bone flap.

**Fig. 1:**
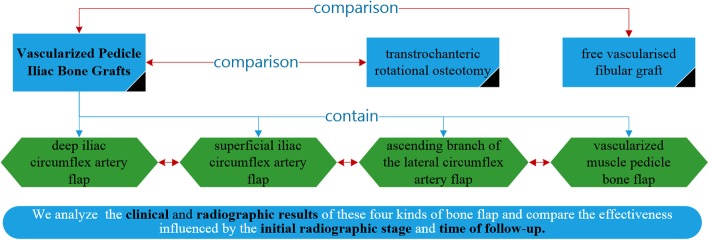
The roadmap of the qualitative analysis

According to the existing literature which could be retrieved from database on the Internet, there have been no review articles to assess the efficacy of VPIBG as a treatment protocol for ONFH. Thus, we performed this systematic review for two purposes: (1) to investigate the clinical and radiographic results of different kinds of VPIBG and (2) to compare the effectiveness of VPIBG influenced by the initial radiographic stage and time of follow-up (Fig. 1).

## Methods

Our systematic review of the literature adhered to the PRISMA guidelines [15, 16]. Methods of the analysis and inclusion criteria were specified in advance.

### Eligibility criteria

#### Types of studies

All types of clinical trials except case report were included in this systematic review. And the language was limited to English.

#### Types of participants

Participants included are those diagnosed with ONFH, who were not performed surgeries on the involved hip except the fixation of femoral neck fracture in traumatic femoral head necrosis. Studies including the majority of juveniles were excluded.

#### Types of intervention

Studies that executed VPIBG were included. If studies examined other treatments, which have been proved to be effective, they will be excluded.

#### Types of outcome measures

The outcome of included trials should contain at least three kinds of measurements: clinical measurements (Harris scores, JOA scores, etc.), radiography measurements, or survival rate of the involved hips.

### Search strategy

Studies were identified by searching electronic databases and scanning reference lists of articles. This search was applied to PubMed, Embase, and the Cochrane Library using the following key terms: femur head necrosis, iliac bone grafting, bone grafting, etc. (Additional file 1: Appendix 1). The last search was run on 13 July 2016.

### Study selection

Two reviewers independently assessed the titles and abstracts of the articles retrieved. For all potentially eligible articles, the full text was obtained and evaluated against the eligibility criteria. Any disagreement between reviewers was resolved by discussion.

### Data collection and analysis

Data extraction included study design, population (patients/hips), and the like by using standardized forms. The VPIBG operation protocols of each study were carefully recorded. The outcome of interest includes clinical evaluation (Harris score, JOA score, etc.), radiographic evaluation (radiographic failure rate), hip survival rate, viability evaluation, and complications. As a result of the different use of scales for clinical evaluation, we analyzed Harris score and other scales respectively, and Harris score was assessed by transforming it into mean difference between pre-operation and last follow-up. In addition, clinical success was defined as Harris score ≥ 80 (Merle d’Aubigne and Postel score ≥ 15, Charnley hip score ≥ 15, HSS ≥ 24); then, clinical success rate could be calculated.

For radiographic evaluation, the kinds of classification system [17] used in studies were converted in accordance with Ficat classification system if possible, for the reason of their similar fundament Table 1. Based on this method, radiographic failure was defined as any lesions progressed to a higher stage from baseline stage except stage I progressed to stage II. And the need for arthroplasty was classified into radiographic failure. Then, the radiographic failure rate was calculated. We collected the date of conversion to arthroplasty or hips requiring secondary operative intervention. Then, the rate of convention to arthroplasty was calculated.

**Table 1:**
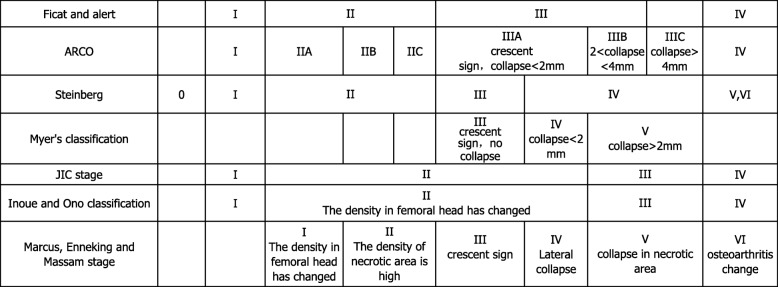
The relationship between stage classifications system of ONFH

For the reason of different follow-up time of these included studies, we categorized the duration of follow-up to short term (less than 6 years) and long term (more than 6 years) because most patients would perform hip replacement surgery in 6 years if the results of VPIBG were not good.

Meta-analysis could not be conducted due to the methodological heterogeneity and the limited number of the available controlled studies. Therefore, we only qualitatively analyze the results extracted from the included studies.

### Risk of bias in individual studies

The methodological study quality was assessed using a checklist for the quality appraisal of case series studies that was developed at the Institute of Health Economics (IHE) [18]. The checklist consisted of 20 criteria. Each study was reviewed by answering “yes,” “partial,” “no,” or “unclear.” “Partial” responses were considered “yes,” and “unclear” was considered “no”; then, we calculate the number of “no” for estimating the risk of bias. A study with 0–2 “no” responses was considered to have a low risk of bias, 3–5 “no” responses a moderate risk, 6–8 a high risk, and ≥ 9 a very high risk of bias [19]. For randomized controlled trial, the Cochrane risk of bias tool was used [20]. The level of evidence of each study was rated on the basis of the Oxford Centre for Evidence-based Medicine—Levels of Evidence (March 2009) (Additional file 1: Appendix 2).

## Results

### Study selection

**Fig. 2:**
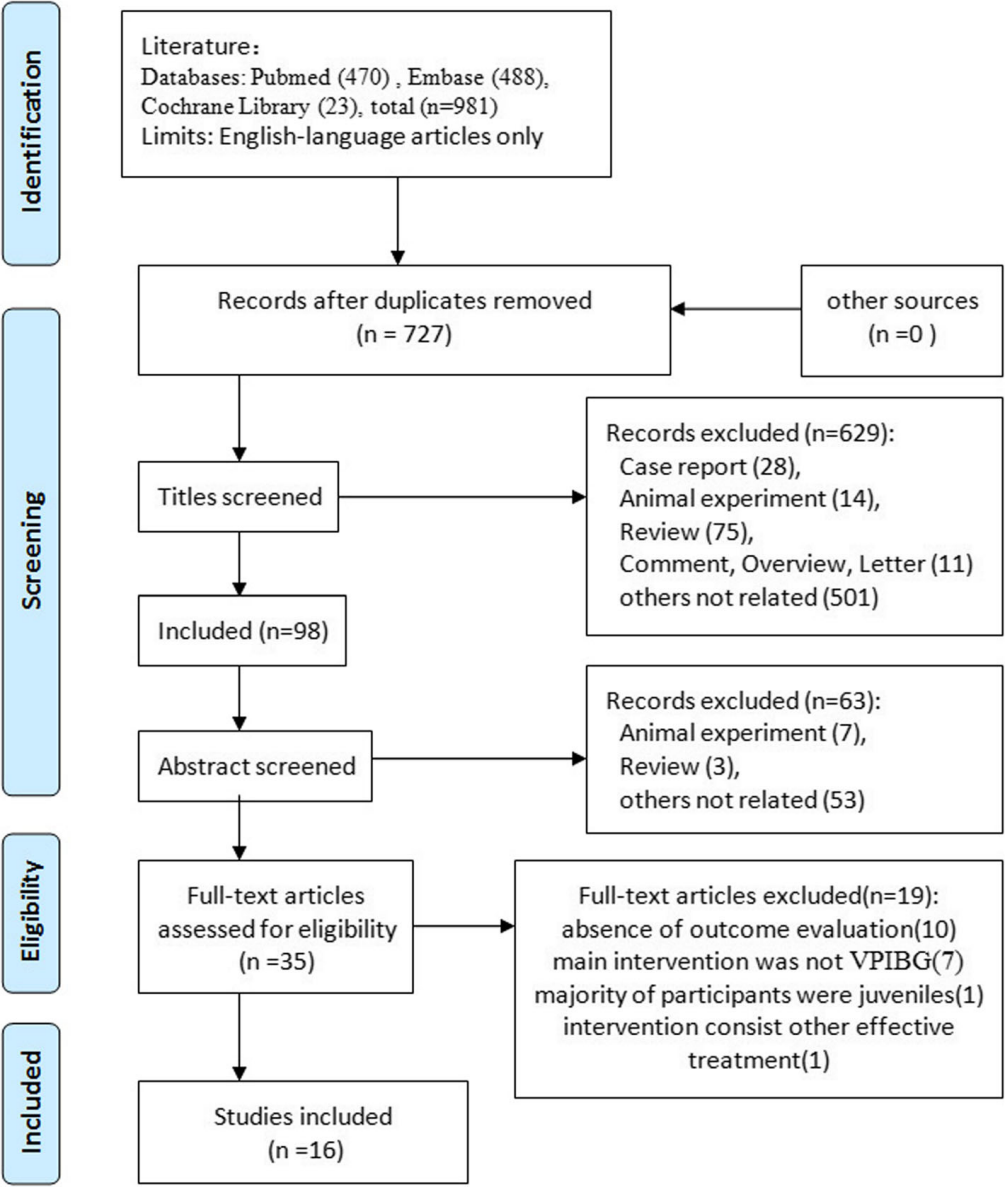
Flow diagram

A total of 16 studies were identified for inclusion in the review. No eligible studies were found by checking the references of location. The detailed process was shown in the flow diagram (Fig. 2).

### Study characteristics

The clinical studies included one randomized control trial (RCT) [21], two case-control studies [22, 23], and 13 case series studies [24–36]. The length of follow-up for the RCTs was 12 months. The two case-control studies had a follow-up that ranged from 3 to 16 years. And the remaining case series were followed up for 2.87 to 16.50 years. The validity of the studies is summarized in Tables 2 and 3 and Additional file 1: Appendix 3.

**Table 2:** Characteristics of the included case series studies

Year/author/region	Study design	Number of patients/hips (follow-up)	Average age (years)	Gender (M/F)	Initial AVN class (hips)	AVN etiology (patients/hips)	Follow-up time (year)/rate of lost to follow-up	Validity of the studies
2016/Chen/China [24]	Case series	55/64	34.1 (22–45)	53/5^a^	ARCO: I (21)/II (35)/III (8)	Alcohol abuse (58)^a^	2.87 (2–4)/5.17%	M
2014/Elmali/Turkey [25]	Case series/retrospective	22/26	36 (16–48)	14/8	ARCO: II (11)/III (15)	Steroid use (13 patients)/alcohol abuse (2)/idiopathic (5)/pregnancy (2)	3 (1.17–5.17)/NA	H
2009/Chen/China [26]	Case series/retrospective	32/33	37 (23–64)	31/1	ARCO: IIIA (26), IIIB (7)	Steroid use (1 patients)/alcohol abuse (26)/idiopathic (5)	5.75 (0.67–13.75)/13.15%	M
2009/Babhulkar/India [27]	Case series/retrospective	31/31	32 (18–52)	26/5	ARCO: IIB (9)/IIIC (22)	Steroid use (12 patients)/alcohol abuse (16)/idiopathic (3)	8/NA	M
2009/Baksi/India [28]	Case series	141/176	35.5 (16–62)	91/61^a^	Ficat and Arlet: I (4)/II (75)/III (97)	Steroid use (56 hips)/alcohol abuse (7)/trauma (40)/idiopathic (83)/gout (1)^a^	16.5 (10–21.5)/ 7.23%	M
2006/Zhao/China [29]	Case series/retrospective	197/226	38 (19–65)	126/71	Ficat and Arlet: II (91)/III (93)/IV (42)	Steroid use (53 patients)/alcohol abuse (14)/trauma (58)/idiopathic (51)/hyperlipidemia (9)/infection (11)/pregnancy (1)	12.5 (6–19)/6.63%	M
2004/Nagoya/Japan [30]	Case series	29/35	35 (17–62)	20/9	ARCO: II (28)/III (7) JIC classification: C1 (17), C2 (18)	Steroid use (14 patients)/alcohol abuse (10)/idiopathic (5)	8.58 (3–17)/NA	H
2001/Eisenschenk/Germany [31]	Case series	82/82	NA	81/21 ^a^	ARCO: I-II (62)/III (1)/IV (19)	Steroid use (12 patients)/alcohol abuse (20)/trauma (22)/diabetes (6)/hyperlipidemia (10)/sickle cell disease (1)/unknown (11)	5 (0.5–10)/19.61%^b^	VH
1997/Hasegawa/Japan [33]	Case series	26/31	38.3 (25–53)	23/3	Inoue and Ono classification: II (28)/IIIA (3) JIC classification: IB (25)/IC (4)/II (4)/ IIIB (1)	Steroid use (6 patients)/alcohol abuse (15)/idiopathic (5)	8 (5–11)/100%	M
1997/Ishizaka/Japan [32]	Case series	24/31	33 (15–66)	16/81	Ficat and Arlet: II (18)/III (13)	Steroid use (17 hips)/alcohol abuse (10)/idiopathic (4)	6 (2–11)/22.58% patient	H
1996/Leung/China [35]	Case series	18/21	32 (24–52)	12/6	Myer’s classification: III (6)/IV (8)/V (7)	Steroid use (7 hips)/alcohol abuse (3)/trauma (3)/idiopathic (8)	NA (2–11)/NA	H
1996/Wassenaar/Netherlands [34]	Case series	9/12	37.5 (14–54)	5/4	Ficat and Arlet: II (5)/III (7)	Steroid use (3 patients)/alcohol abuse (1)/trauma (3)/chemotherapy (1)/sickle cell disease (1)	4.17/30.77%	VH
1993/Iwata/Japan [36]	Case series	19/23	39 (25–53)	17/2	JIC stage: II (20), III (3) JIC classification: I-C (19)/II (2)/III-B (1)	Steroid use (3 patients)/alcohol abuse (13)/idiopathic (3)	2.91 (1–6)/100%	H

*L* low risk of bias, *M* moderate risk, *H* high risk, *VH* very high risk, *NA* not available

^a^Include participants who are lost to follow-up

^b^Lost to follow-up cases include participants converted to arthroplasty

**Table 3:** Characteristics of included control studies

Year/author	Study design	Number of patients/hips (follow-up)		Average age (years)		Gender (M/F)		Initial AVN class (hips)		AVN etiology (patients/hips)		Follow-up time (years) /rate		Validity of the studies
		TIG	Other	TIG	Other	TIG	Other	TIG	Other	TIG	Other	TIG	Other	
2016/Zhao/China^a^ [21]	RCT	23/23	25/25	30 ± 7	33 ± 8	14/9	15/10	ARCO: IIA (4)/IIB (5)/IIC (4)/IIIA (4)/IIIB (4)/IIIC (2)	ARCO: IIA (5)/IIB (5)/IIC (3) IIIA (5)/IIIB (4)/IIIC (3)	Steroid (7 patients)/alcohol (7)/others (9)	Steroid (9 patients)/alcohol (5)/others (11)	1/100%	1/100%	Additional file 1: Appendix 2
2006/Yen/China^b^ [22]	Case-control	33/39	22/22	40 (26–63)	38 (28–52)	30/3	20/2	Steinberg: II (11)/III (12)/IV (16)	Steinberg: II (4)/III (11)/IV (7)	Steroid (1 patient)/alcohol (15)/idiopathic (17)	Steroid (1 patient)/alcohol (12)/idiopathic (9)	> 4/NA	> 3/NA	Additional file 1: Appendix 2
2003/Hasegawa/Japan+^c^ [23]	Case-control	26/31	63/77	37.9 (25–53)	39 (19–64)	23/3	44/19	Ficat and Arlet: II (28)/III (3) JIC classification: IB (1)/IC (25)/II (4)/III (1)	Ficat and Arlet: II (34)/III (43) JIC classification: IB (3)/IC (62)/II (12)	Steroid (8 hips)/alcohol (16)/idiopathic (7)	Steroid (46 hips)/alcohol (24)/idiopathic (7)	13 (10–15)/3.84%	7 (5–11)/3.07%	Additional file 1: Appendix 2

*TIG* target intervention group, *other* other treatments as a control group for the target intervention group, *NA* not available

^a^TIG refers to the Mg screw group (vascularized bone grafting fixed by Mg screws), and other treatments refer to vascularized bone grafting without fixation as a control group

^b^TIG refers to vascularized iliac pedicle bone grafting, and other treatments refer to free vascularized fibula bone grafting

^c^TIG refers to vascularized iliac pedicle bone grafting, and other treatments refer to transtrochanteric rotational osteotomy

The included studies comprised of 877 participants (1011 hips). The main inclusion criteria entailed adults (mean age varied from 30 to 40), who suffered from the following etiology: trauma, steroid use, alcohol abuse, idiopathic, etc.

The severity of AVN was classified using diverse grading systems. Seven of these studies [21, 24–26, 28, 30, 31] utilized the Association Research Circulation Osseous (ARCO) classification. Five studies [23, 27, 29, 32, 34] used the Ficat classification. And the others used Steinberg, JIC, Inoue and Ono classification, Myer’s classification, respectively (Table 2). And Table 1 summarizes the relationship between these classifications based on their similar fundament.

Five studies [22, 23, 27, 33, 36] described the indications of VPIBG. Majority of the indications reported share the same concept, and we synthesized into the following items: (1) pain and discomfort around the hip and the limitation of movement of the hip [27], (2) collapse of femoral head was less than 2 mm [33, 36], (3) IC or II class femoral head according to JIC classification [23, 33], and (4) femoral heads collapsing more than 5 mm were not recommended to perform VPIBG [36]. Femoral heads classified into ARCO IIIB or IIIC stage were also considered as the indications in 2 studies [22, 23].

The nutrient vessels for iliac bone flap used by included studies cover DICA [23, 25, 26, 28, 30–33, 35, 36], SICA [23, 33, 36], and ALCA [21, 29]. Two studies [24, 27] used tensor fascia lata and sartorius muscle-pedicle bone flap respectively. And the kind of flap used was not mentioned in the remaining 2 studies [22, 34] (Tables 4 and 5).

**Table 4:** The intervention characteristics of case series studies

Year/author	Indications/contraindication	Kinds of pedicle bone flap	Size of bone flap (cm)	Duration of the operation (minutes)	Postoperative management	Additional note
2016/Chen [24]	NA	Sartorius muscle-pedicle bone flap	4 × 2 × 2.5	65 (45–90)	Partial weight-bearing walking at 8 to 12 weeks, and further advice was subject to radiographic evidence after 3 to 6 months of operation	Bone flap was fixed by absorbable screw
2014/Elmali [25]	NA	Deep circumflex iliac artery pedicle bone flap	6 × 2 × 2	210 (168–258)	The hip was positioned in 20° flexion for 3 weeks, full weight-bearing walking at 6 months	–
2009/Babhulkar [28]	NA	Deep circumflex iliac artery pedicle bone flap	NA	NA	The limb was kept in 20° abduction and 30° flexion and 10° of internal rotation, and allowed free movements in bed after 15 days, non-weight-bearing after 4–6 weeks, partial weight-bearing after 10 weeks and full weight-bearing after 14–16 weeks	–
2009/Chen [26]	NA	Deep circumflex iliac artery pedicle bone flap	5 × 1.5	NA	Toe-touch weight-bearing for 6 weeks, then partial weight-bearing for 6 months and full weight-bearing was allowed after 6 months	–
2009/Baksi [27]	Pain and discomfort around the hip, limitation of movement	Tensor fascia lata muscle-pedicle bone flap	2.5 × 2.5	NA	Partial weight-bearing was allowed after 5 weeks, and full weight-bearing was allowed after 4–5 months	Bone flap was fixed by Vicryl thread
2006/Zhao [29]	NA	Ascending branch of the lateral femoral circumflex artery pedicle bone flap	5 × 3	90 (55–120)	Bed rest with traction was used for 45 days postoperatively, progressive weight-bearing was permitted after the graft incorporated with the host area. Full weight-bearing was achieved by 6 months	–
2004/Nagoya [30]	NA	Deep circumflex iliac artery pedicle bone flap	NA	NA	Bed rest was prescribed for 2 weeks. Weight-bearing exercise started 4 weeks after surgery, followed by partial weight-bearing for the next 6 months	Bone flap was fixed by AO screw
2001/Eisenschenk [31]	NA	Deep circumflex iliac artery pedicle bone flap	6 × 2	NA	The hip was positioned in 20° after operation, weight-bearing was not allowed for 6 months after operation	–
1997/Hasegawa [33]	Indications: Collapse < 2 mm JIC IC, II type	Superficial circumflex iliac artery pedicle bone flap (deep circumflex iliac artery was used in 8 hips)	5 × 1.5	NA	Ambulation was not permitted for 3 weeks, partial weight-bearing was encouraged after 12 weeks, full weight-bearing was permitted after 24 weeks.	Hyperbaric oxygen therapy was conducted on 11 patients
1997/Ishizaka [32]	NA	Deep circumflex iliac artery pedicle bone flap	5 × 2 × 2	150	Patients were kept non-weight-bearing for 6 months, followed by partial weight-bearing with for the next 6 months.	–
1996/Leung [35]	NA	Deep circumflex iliac artery pedicle bone flap	NA	NA	The hip was kept in 30″ flexion and neutral rotation for 1 week. Hip motion was encouraged. Bed rest was continued for 3 to 4 weeks. Non-weight-bearing walking was allowed• after 4 weeks, and weight-bearing was delayed until 8 weeks	–
1996/Wassenaar [34]	NA	NA	NA	NA	NA	–
1993/Iwata [36]	Indications: JIC I/II stage Contraindication: collapse > 5 mm	Superficial circumflex iliac artery pedicle bone flap (deep circumflex iliac artery was additionally used in 2 cases)	NA	NA	Active exercise in the second week, exercise in the pool in the fourth week, one-third weight-bearing in the 12th week, and full weight-bearing in the 24th week	Hyperbaric oxygen therapy was conducted on 13 patients

*NA* not available

**Table 5:** The intervention characteristics of the control studies

Year/author	Indications/contraindication	Kinds of pedicle bone flap		Size of bone flap (cm)		Duration of the operation (minutes)		Postoperative management		Additional note	
		TIG	Other	TIG	Other	TIG	Other	TIG	Other	TIG	Other
2016/Zhao^a^ [21]	NA	Ascending branch of the lateral femoral circumflex artery pedicle bone flap		5 × 3	5 × 3	NA	NA	NA	NA	Bone flap was fixed by absorbable screw	–
2006/Yen^b^ [22]	Steinberg II, III, IV Stage	NA	NA	NA	NA	210	420	NA	NA	–	–
2003/Hasegawa^c^ [23]	TIG: Ficat II stage and JIC IC or II type Other: Ficat III stage or above, and JIC IC or II type, and the necrosis area < 36% in frog lateral view	Superficial circumflex iliac artery pedicle bone flap	–	NA	–	NA	NA	Exercises were started in the third week after the operation. At 12 weeks partial weight-bearing (10 kg) and at 24 weeks full weight-bearing were allowed.		–	–

*TIG* target intervention group, *other* other treatments as a control group for target intervention group, *NA* not available

^a^TIG refers to the Mg screw group (vascularized bone grafting fixed by Mg screws), and other treatments refer to vascularized bone grafting without fixation as a control group

^b^TIG refers to vascularized iliac pedicle bone grafting, and other treatments refers to free vascularized fibula bone grafting

^c^TIG refers to vascularized iliac pedicle bone grafting, and other treatments refer to transtrochanteric rotational osteotomy

As to the average time of operation, sartorius muscle-pedicle bone flap grafting costs the shortest [24], for only 45 to 90 min. DICA bone grafting takes 2.8 to 4.3 h [25, 32], and ALCA bone grafting 0.92 to 2 h [29]. Several studies additionally fixed grafted flap by biodegradable Mg screw [21], absorbable screws [24], AO screws [30], and Vicryl thread [27].

### Outcome

Across the included trials, the outcome was measured by various methods including clinical evaluation, radiographic evaluation, hip survival rate, and complications (Tables 6, 7, and 8). We summarized and analyzed the outcomes not mentioned in the table on the basis of different kinds of VPIBG respectively.

**Table 6:** The outcome of case series studies

Year/author	Clinical evaluation		Clinical evaluation based on the Ficat stage classification (mean difference/clinical success rate)				Radiographic evaluation (radiographic failure rate)	Complications/rate of complications
	Mean difference^a^ (scale used)	Clinical success rate^b^	I	II	III	IV		
2016/Chen [24]	31.06 (HHS)	79.68%	32.22/90.47%	31.2/77.14%	26.13/62.50%	–	NA	NA
2014/Elmali [25]	30.8 (HHS)	69.23%	–	NA	NA	–	34.62% II (36.36%) III (33.33%)	2 patients suffering from obesity with serious drainage for 1 week 2/22 (9.09%)
2009/Babhulkar [28]	28.19 (HHS)	58.06%	–	29.88/62.50%	27.61/56.5%	–	6.45%	Superficial infection at the operative site (1 patient) 3.22%
2009/Chen [26]	8.92 (HHS)	NA	–	–	NA	–	100%	NA
2009/Baksi [27]	4.06 (HSS)	85.79%	1/100%	4.8/92%	3.6/80.4%	–	9.65%	Superficial wound infection (9 hips), terminal limitation of hip movements (20), persistence of painless limp (16) 25.57%
2006/Zhao [29]	38 (HHS)	86.28%	–	NA/96%	NA/90%	NA/57%	28.76%	Deep venous thromboses (4 patients), meralgia paresthetica (3), secondary wound healing (9) 8.12%
2004/Nagoya [30]	17.35 (JOA)	NA	–	NA	NA	–	NA	Damage of cutaneus femoris lateralis nerve (10 patients) 34.48%
2001/Eisenschenk [31]	NA (HHS)	86.6%	NA	NA	NA	–	48.89%	Deep thrombosis of the femoral vein (2 patients), damage of cutaneus femoris lateralis nerve (7), abdominal weakness without evidence of hernia (5), secondary wound healing (2) 16/82 (19.51%)
1997/Hasegawa [33]	21 (HHS)	63.33%	–	NA	NA	–	NA	Secondary wound healing (3 hips), damage of cutaneus femoris lateralis nerve (8) 36.67%
1997/Ishizaka [32]	2.2 (Merle d’Aubigne and Postel score)	77%	–	NA	NA	–	48.38%	NA
1996/Leung [35]	5.06 (Charnley hip scoring system)	77.78%	–	–	NA	–	NA	NA
1996/Wassenaar [34]	28 (HHS)	NA	NA	NA	NA	–	41.66%	NA
1993/Iwata [36]	9.7 (JOA)	NA	NA	12.46/NA	3.34/NA	–	34.78%	Secondary wound healing (2 hips), damage of cutaneus femoris lateralis nerve (5) 30.43%

*NA* not available, *HHS* Harris hip score, *HSS* Hip rating system of the Hospital for Special Surgery, *JOA* Japan Orthopaedic Association hip score

^a^The mean difference was defined as the difference between the mean score of pre-operation and final follow-up

^b^Clinical success was defined as the Harris score ≥ 80 (Merle d’Aubigne and Postel score ≥ 15, Charnley hip score ≥ 15, HSS ≥ 24)

**Table 7:** The outcome of control studies

Year/author	Clinical evaluation				Clinical evaluation based on the Ficat stage classification (mean difference/clinical success rate)								Radiographic evaluation (radiographic failure)		Complications /rate of complications	
	Mean difference (scale used)		Clinical success rate		TIG				Other				TIG	Other	TIG	Other
	TIG	Other	TIG	Other	I	II	III	IV	I	II	III	IV				
2016/Zhao^a^ [21]	26.08 (HHS)	22.53 (HHS)	95.65%	76.00%	–	21.16/100%	32.37/90%	–	–	17.34/92.31%	28.07/58.33%	–	8.7%	24%	NA	NA
2006/Yen^b^[22]	2 (Charnley Functional Hip Score)	3 (Charnley Functional Hip Score)	NA	NA	–	NA	NA	NA	–	NA	NA	NA	43.59%	18.18%	Mild wound-edge necrosis (2), damage of cutaneus femoris lateralis nerve (5), inguinal protrusion (1) 24.24%	Claw toe (1) 4.54%
2003/Hasegawa ^c^ [23]	14 (HHS)	18 (HHS)	58%	68%	–	NA	NA	–	–	NA	NA	–	NA	NA	Mild wound-edge necrosis (3), damage of cutaneus femoris lateralis nerve (8) 42.31%	Early deep infection (1), trochanteric fracture (5), pseudarthrosis (1) 11.11%

*TIG* target intervention group, *other* other treatments as a control group for the target intervention group, *NA* not available

^a^TIG refers to the Mg screw group (vascularized bone grafting fixed by Mg screws), and other treatments refer to vascularized bone grafting without fixation as a control group

^b^TIG refers to vascularized iliac pedicle bone grafting, and other treatments refer to free vascularized fibula bone grafting

^c^TIG refers to vascularized iliac pedicle bone grafting, and other treatments refer to transtrochanteric rotational osteotomy

**Table 8:** The evaluation of hip survival

Study	Rate of convention to arthroplasty (arthroplasty/total hips)	Method of report in original studies	Survival rate (based on the definition in original studies)	Definition of hip-preservation failure in individual study	Survival rate based on stage
2016/Zhao^a^ [21]	0%	NA	NA	NA	NA
2016/Chen [24]	14.06% (9/64)	Survival curve	81.25%	The collapse of the femoral head was larger than 4 mm, with significant osteoarthritis; or hip replacement	NA
2014/Elmali [25]	19.23% (5/26)	Reported cases of arthroplasty	NA	NA	NA
2009/Chen [26]	75.76% (25/33)	Survival curve	24.24%	Conversion to replacement arthroplasty	ARCO IIIB, 0% ARCO IIIA, 30.77%
2009/Babhulkar [28]	3.23% (1/31)	Reported cases of arthroplasty	NA	NA	NA
2009/Baksi [27]	Fail to recalculate	Survival curve	83.97%	Radiological deterioration, resulting in the reduction of the clinical HSS score to below 20, requiring subsequent operative intervention	Ficat II, 91% Ficat III, 82%
2006/Yen^b^ [22]	10.26% (4/39)	Reported cases of arthroplasty	10.26%	Conversion to replacement arthroplasty	Steinberg III, 75% Steinberg IV, 93.75%
2006/Zhao [29]	6.19% (14/226)	Reported cases of arthroplasty	93.81%	Conversion to replacement arthroplasty	NA
2004/Nagoya [30]	NA	NA	NA	NA	NA
2003/Hasegawa^b^ [23]	6.45% (2/31)	Survival curve	5Y, 85% 10Y, 67%	The development of symptoms requiring arthroplasty or a Harris hip score of less than 70 points as the endpoint	NA
2001/Eisenschenk [31]	8.89% (8/90)	Reported cases of arthroplasty	NA	NA	NA
1997/Hasegawa [33]	3.23% (1/31)	Survival curve	Satuation 1: 3Y, 70%; 5Y, 60% satuation 2: 3Y, 60%; 5Y, 50%	Satuation 1: overall clinical score of less than 70 points or conversion to an endoprosthesis satuation 2: radiographic stage further than stage III-B (collapse by more than 5 mm)	NA
1997/Ishizaka [32]	9.68% (3/31)	Reported cases of arthroplasty	NA	NA	NA
1996/Leung [35]	4.76% (1/21)	Reported cases of arthroplasty	NA	NA	NA
1996/Wassenaar [34]	8.33% (1/12)	Reported cases of arthroplasty	NA	NA	NA
1993/Iwata [36]	4.35% (1/23)	Reported cases of arthroplasty	NA	NA	NA

*NA* not available

^a^The evaluation combined two groups

^b^The evaluation of the VPIBG group

### Clinical evaluation

Clinical evaluation was described using the following methods: Harris hip score [24–26, 28, 29, 31, 33, 34], Japan Orthopaedic Association hip score (JOA score) [30, 36, 37], hip rating system of the Hospital for Special Surgery (HSS score) [27], Merle d’Aubigne and Postel score (MP score), and Charnley hip scoring system (Charnley score) [22, 35]. Mean difference and clinical success rate were calculated then summarized in Tables 6 and 7.

#### DICA pedicle iliac bone grafting

As is shown in Tables 4 and 5, most studies used this surgical method relatively got satisfactory therapeutic results. However, Babhulkar [28] reported that only 13 (56.25%) stage III hips got a clinical success result. Chen et al. [26] reported that the average Harris score in the last follow-up was only 64.85 in stage III hips. These 2 studies both indicated a poor result in stage III hips. But Leung’s studies [35] suggested a satisfying result for a clinical success rate of 77.78% in stage III hips. Therefore, the efficiency of this surgical method between different stage hips needs further research. Ishizaka et al. [32] found the therapeutic effect was related to the position of the necrotic area, for the reason of a higher clinical success rate (85%) in medial necrotic hips than lateral necrosis which only got a clinical success rate of 72%. Eisenschenk et al. [31] compared the therapeutic effect in different follow-up time and indicate a better clinical result in the early outcome.

#### SICA pedicle iliac bone grafting

Hasegawa et al. [33] used this surgical procedure resulting in a medium clinical success rate (63.33%). This research group previously compared the outcome in different follow-up time and primary stage of ONFH in 1993. And the results have no difference in the short-term follow-up. Furthermore, the JOA score of the hips in early stage got a greater promotion. In 2003, a controlled study [23] was performed by them and suggested a higher clinical success rate in the TRO group than in the VPIBG group, and the clinical success rate decreased with an increase in follow-up time.

#### ALCA pedicle iliac bone grafting

Zhao et al. [29] executed this surgical method, and the clinical data was calculated and resulted in a poor result in stage IV hips. Furthermore, this research group performed the first randomized control trial (RCT) [21], which did a comparison of outcomes between two groups performing this surgery with or without flap fixation using biodegradable Mg screw. The Harris hip score exhibited a mild increase of clinical function at 12 months compared with that at 6 months after the operation. As to the clinical success rate, stage II hips exhibited a better result than stage III, especially the control group.

The two researches above both suggested better clinical results in the early stage of ONFH, but there exist marked variation in the clinical evaluation of stage III hip across two studies.

#### Muscle-pedicle iliac bone grafting

Baksi et al. [27] performed VPIBG using the tensor fascia lata pedicle bone flap and additionally fixed the embedded flap with Vicryl thread and observed a poorer result in the long-time follow-up and a better result in an early stage. A study performed by Chen et al. [24] got similar results by using the sartorius muscle-pedicle bone flap. Yen et al. [22] made a contrast between VPIBG and free vascularized fibular graft (FVFG), but did not report the type of iliac flap. And there was no obvious difference of clinical results between these two groups at the final follow-up.

### Radiographic evaluation

The following methods were used for classifying the stage of ONFH: ARCO classification [21, 24–26, 28, 30, 31], Ficat and Arlet classification [23, 27, 29, 32, 34], Myer’s classification [35], JIC stage classification [36], and Inoue and Ono classification [33]. According to the relation described in Table 1, these classification systems were transformed into Ficat classification if possible. The radiographic failure rate is calculated and summarized in Tables 6 and 7.

#### DICA pedicle iliac bone grafting

Majority of the studies resulted in a radiographic failure rate of less than 50%. Elmali et al. [25] evaluated the radiographic result of stage II and III hips and inferred similar radiographic results across stage II and III hips. Ishizaka et al. [32] also observed no significant difference between stages II and III and reported that the necrotic hips of lateral type exhibit a higher possibility of collapse compared with those of medial type. These results coincide with the clinical evaluation. Babhulkar et al. [28] reported a low radiographic failure rate both in stage II and III hips.

In contrast, two studies reported poor radiographic results particularly in stage III hips. Chen et al. [26] reported a radiographic failure rate of 100%. Nagoya et al. [30] found more stage III hip progressed to collapse more than 2 mm compared with stage II. And the results also indicate a better result of type C-1 hips. Furthermore, this study found that the insertion of the pedicle bone close to the anterolateral normal subchondral bone of the femoral head gave better results in terms of preventing the collapse of the femoral head.

#### SICA pedicle iliac bone grafting

The study performed by Iwata et al. [36] evaluates the radiographic results according to JIC classification. For the reason of small size of stage III hips, the difference of radiographic results between stage II and stage III hips has no obvious meaning. This research group evaluates a similar study performed in 1997 [33], and according to the number of stage II hips at different follow-up time reported by the two studies above, more stage II hips were observed to progress to a higher stage as time of follow-up grows, indicating a better result in short term than in long term of follow-up, which was further proved by the control study performed by the same research team in 2003 [23]. Furthermore, this controlled study also reported a higher rate of stage II hips that progressed to a higher stage in the VPIBG group compared with the transtrochanteric rotational osteotomy (TRO) group.

#### ALCA pedicle iliac bone grafting

Zhao et al. [29] estimated the radiographic failure rate and indicated a better result in early-stage hips. In the RCT performed in 2016 by the same research team [21], the fixation of the implanted bone flap using Mg screw suggested a lower radiographic failure (8.70%) compare with the control group (24.00%), primarily because of the low rate of flap displacement.

#### Muscle-pedicle iliac bone grafting

Chen et al. [24] performed VPIBG using the sartorius muscle-pedicle bone flap and Baksi et al. [27] using tensor fascia lata pedicle bone flap. Both studies suggested a better result in the early-stage hips.

Wassenaar et al. [34] and Yen et al. [22] reported no information of the iliac flap type used. The former found a higher radiographic failure rate in Ficat stage II hips (40%) than that in stage III hips (28.57%). Whereas this result made little sense due to the small sample size consisting only 5 stage II and 7 stage III hips, the latter compared the difference of radiographic results in the VPIBG and FVFG groups and reported a higher radiographic failure rate in the VPIBG group than in the FVFG group (43.59% versus 18.18%).

#### Survival rate

The included studies all reported the result of progression to arthroplasty or secondary surgery on involved hips after VPIBG except one study [30]. They either reported the survival rate using a Kaplan-Meier survival curve [24, 26, 27, 33, 35] or the number of hips progressed to arthroplasty. Some researches defined the survival rate or failure of hip preservation in their own ways. The results are summarized in Table 8.

As to the difference between short-term efficiency and long-term efficiency, Hasegawa et al. [33] reported the efficiency was mildly better at 3 years than 5 years of follow-up. This research team performed a longer time of follow-up in 2003 [23] and also suggested the efficacy in the short term was markedly better than that in the long term.

Baksi et al. [27] and Chen et al. [26] reported a poorer result in stage III compared with that in stage II, particularly in stage IIIB hips, of which the survival rate was 0%. In contrast, the research of Yen et al. [22] reported a higher survival rate in Steinberg stage IV hips (equal to ARCO IIIB and IIIC) compared with that in stage III (equal to ARCO IIIA), arising a contradiction with the study of Chen et al. [26]. However, the conclusion was unrepresentative due to the small sample size.

Two included control studies [21–23] all suggest a lower rate in the FVFG and TRO group than in the VPIBG group.

### Viability evaluation of implanted flap

Chen et al. [26] execute MRI or Tc-99 m single-photon emission computed tomography for viability evaluation and found the graft was viable in 24 hips (96%). Super selective angiographies were conducted by Eisenschenk et al. [31] and indicated perfusion of the transplants in 35 patients (83.3%). Bone grafts in the study of Wassenaar et al. [34] all demonstrated sum rounding sclerosis and fusion to the surrounding bone by radiographic evaluation. The three studies above all suggested the viability of the implanted iliac flap. On the other hand, Iwata et al. [36] found that 15 hips (65.22%) examined by photon emission computed tomography resulted in good viability. This team confirmed this conclusion again in 1997 [33].

### Complications

Ten researches [22, 23, 27–32, 36] reported complications summarized in Tables 6 and 7, which include superficial wound infection or necrosis, deep thrombosis of the femoral vein, and the like. And the occurrence rate of complications ranges from 8.12% (16/197 patients) to 42.31% (11/26 patients).

Compared with other operations such as FVFG and TRO, the complications of VPIBG has a higher occurrence rate but much slighter. Yen et al. [22] reported that one patient who accepted FVFG suffered from claw toe probably due to the wake of peroneus muscle contracture. In the study of Hasegawa et al. [23], which performed TRO to 63 patients, early deep infection occurred in one patient, trochanteric fracture in five, and pseudarthrosis in one, indicating the possibility of severe complications in some extent.

## Discussion

On the basis of our retrieved result, this systematic review first summarizes and evaluates the VPIBG treatment as a hip-preserving surgery for ONFH. This protocol is suitable for the early stage of necrosis (before collapse) [38]. By opening a bone window at the junction of femoral head and neck, necrotic tissues could be excised more thoroughly than the approach via tunnels in the femoral neck [39]. Then, vascularized iliac bone flap and cancellous bone were implanted into the cavity, resulting in the mechanical support for the subchondral bone to prevent collapse. Furthermore, a favorable environment for bone induction and formation was created by the reconstruction of blood supply in the necrotic area.

Our systematic review includes case series study in 1993 and also the randomized controlled trial in recent years. Due to the large time span and national difference, the methods used to evaluate the study outcomes and severity of ONFH vary significantly. Therefore, we unify the outcome indicator based on characteristics of scales and classification system in order to compare the difference of outcome across studies. As to the analysis of results, we divided included studies into several subgroups due to the heterogeneity of their characteristics in order to recognize the difference of effectiveness influenced by the initial radiographic stage and follow-up time.

Based on the above literature survey, the effectiveness of VPIBG correlates with the stage of ONFH when the operation was conducted. Femoral heads before the collapse of subchondral bone (appearance of the crescent sign) generally get better results in clinical evaluation than those after this time point. However, there exist some differences across included studies, such as the different clinical success rate in stage III hips reported by two individual studies [26, 35]. This may correlate with the subjectivity of the clinical evaluation or sample size. On the other hand, the radiographic evaluation also demonstrates the conclusion which the clinical results have implied. And similarly, there exist some studies that indicated no obvious difference between collapsed and pre-collapsed hips [28, 31, 32]. And these results may be affected by the short follow-up time and small sample size included by researches. In addition, studies demonstrated that surgical efficiency was related to the position of the necrotic area, for instance, the medial type of necrosis got a much better result compared with the lateral type [32], and treatment efficiency in JIC type C1 hips was better than that in type C2 hips [30]. The analysis of survival rate further approves these two conclusions above.

Some studies evaluate the clinical results at different time points of follow-up, generally suggesting a better result in the short term. Study also suggested no obvious difference on clinical efficiency within 3 to 5 years [21]. There exist two studies, however, executed by one research team in different time quantum, which demonstrated that the clinical success rate of long-term follow-up (12.5 years) preceded that of short-term follow-up (86.28% versus 76.00%) [21, 29]. Additional examinations were executed to testify the viability of the implanted bone flap, indicating that the majority of the flaps maintained viability. Complications showed a high occurrence rate relatively, but the majority were slight and rarely affected clinical efficacy.

Heterogeneities exist in follow-up time, sample size, result evaluation, and interventions. Single arteriovenous pedicle provides relative abundant blood supply compared with muscles, but should be rerouted to the recipient site, and kinking, compression, or overstretching of the vascular pedicle is possible. These disadvantages ultimately result in a long operation time and high risk of failure. In contrast, the muscles contain numerous vascular communications, which were well protected within the muscle bed, to nourish the flap. And then, muscle-pedicle bone grafting procedures appear relatively easier technically. Due to the uncertainty of nourishing vessels, however, the blood supply of bone flap also remains uncertain. Some studies additionally fixed the implanted flap. In addition, the position of the flap in the femoral head also affected the results. The above mentioned all indicated that heterogeneities existing in the intervention of the included studies further lead to the heterogeneities of results.

As to the characteristics of participants in individual studies, some indicated a strong possibility of a collapse in the lateral type of necrosis compared with the medial type [32], and JIC type C-2 necrosis also resulted in a higher collapse rate compared with type C-1 [30]. However, the majority of included researches did not report the position of the necrosis area in the femoral head, which also indicated a potential heterogeneity affecting the results between studies. Besides, etiology and severity of ONFH also exist as heterogeneities leading to the heterogeneities of results across the included studies.

Compared with VPIBG, FVFG gave a lower possibility of collapse [22]. This probably associated with the stronger biomechanical support provided by fibula. The incision of a hip joint capsule in VPIBG procedure may further injure the blood supply of the femoral head. And the FVFG procedure avoids this disadvantage. Nevertheless, the incomplete excision of necrotic tissues, prolonged operation time, need of microvascular technique, donor site morbidity, and possibility of heterotopic ossification probably cause a poor efficiency and high complication rate. Femoral trochanter flap grafting was also used as a similar hip-preserving approach, generally using the transverse branch of the femoral circumflex artery as the nutrient vessels [40, 41], and the channel of bone grafting is similar to VPIBG. VPIBG combined with other approaches, such as TRO [42–44] and tantalum rod support [45], to achieve a better clinical efficiency. TRO could transfer the weight-bearing area of the femoral head to the intact area. And tantalum rod primarily reinforces the mechanical support to the subchondral bone, finally researches a similar efficacy of FVFG, and avoids the deficiency of the FVFG mentioned above.

Although we have strictly formulated uniform standards of result evaluation, our systematic review still has limitations: ① We cannot analyze other factors like patient’s comorbidities that potentially influence efficacy of VPIBG, because the included studies rarely considered this factor; ② Due to the lack of RCT or case-control studies, the efficacy of VPIBG cannot be evaluated accurately; ③ The quality of the recommendations in this systematic review is relatively low, for the reasons of heterogeneity among the included studies. Finally, we only executed a descriptive analysis for this systematic review, instead of a quantitative analysis.

## Conclusion

In combination with what has been discussed above, the VPIBG gets a better clinic response in ONFH before the appearance of a crescent sign through X-ray graphs compared with the femoral head after the collapse. And a better result is gotten in the short term compared with a long-term follow-up, but there exists no obvious difference on clinic efficiency within 3 to 5 years. The fixation of the implanted iliac bone flap increases the clinical effect. As for the complications of VPIBG, the majority were slight and rarely affected clinical efficacy. Nevertheless, on account of the lack of high-quality research presented and inter-study heterogeneity, these conclusions need the support of further research, which includes (1) more control studies, especially RCTs, to verify the clinical utility by comparing with other treatments; (2) unified result evaluation system to reduce heterogeneity; and (3) operation approach which needs to be further standardized, in order to find out the optimization-specific protocol of VPIBG.

## Additional file

Additional file 1: **Appendix 1**. Search strategy: PubMed. Appendix 2. Oxford Centre for Evidence-based Medicine—Levels of Evidence (March 2009). Appendix 3. Quality evaluation of included studies case series studies. (DOCX 33 kb)

## Data Availability

Data sharing is not applicable to this article as no datasets were generated or analyzed during the current study.

## Abbreviations

ALCA: Ascending branch of the lateral circumflex artery
ARCO: Association Research Circulation Osseous
DICA: Deep iliac circumflex artery
JIC: Japanese investigation criteria
ONFH: Osteonecrosis of the femoral head
RCT: Randomized controlled trial
SICA: Superficial iliac circumflex artery
VPIBG: Vascularized pedicle iliac bone grafts
