# Supplementary material for: Vascularized pedicle iliac bone grafts as a hip-preserving surgery for femur head necrosis: a systematic review
Source: J Orthop Surg Res. 2019 Aug 27;14:270. doi: 10.1186/s13018-019-1262-2 (PMC6710879; doi:10.1186/s13018-019-1262-2)
Supplement: Supplementary file 1 — Appendix 1. Search strategy: PubMed. Appendix 2. Oxford Centre for Evidence-based Medicine—Levels of Evidence (March 2009). Appendix 3. Quality evaluation of included studies case series studies. (DOCX 33 kb) [file 13018_2019_1262_MOESM1_ESM.docx]

**Appendix 1. Search strategy: Pubmed**

| **Search** | **Query** |
| --- | --- |
| #1 | Femur Head Necrosis[MeSH Terms] |
| #2 | Femur Head Necroses |
| #3 | Femur Head Aseptic Necrosis |
| #4 | Femur Head Avascular Necrosis |
| #5 | Femoral Head Ischemic Necrosis |
| #6 | Femoral Head Necroses |
| #7 | Femoral Head Aseptic Necrosis |
| #8 | Femur Head Ischemic Necrosis |
| #9 | Femoral Head Avascular Necrosis |
| #10 | #1 OR #2 OR #3 OR #4 OR #5 OR #6 OR #7 OR #8 OR #9 |
| #11 | Bone Transplantation[MeSH Terms] |
| #12 | bone graft* |
| #13 | vascularized bone graft* |
| #14 | vascularized iliac bone graft* |
| #15 | vascularized ilium bone graft* |
| #16 | #11 OR #12 OR #13 OR #14 OR #15 |
| #17 | #10 AND #16 |
| #18 | #10 AND #16 Filters: English |

**Appendix 2.** Oxford Centre for Evidence-based Medicine – Levels of Evidence (March 2009)

| **Level** | **Therapy / Prevention, Aetiology / Harm** | **Prognosis** | **Diagnosis** | **Differential diagnosis / symptom prevalence study** | **Economic and decision analyses** |
| --- | --- | --- | --- | --- | --- |
| 1a | SR (with homogeneity*) of RCTs | SR (with homogeneity*) of inception cohort studies; CDR”  validated in different populations | SR (with homogeneity*) of Level 1 diagnostic studies; CDR”  with 1b studies from different clinical centres | SR (with homogeneity*) of prospective cohort studies | SR (with homogeneity*) of Level 1 economic studies |
| 1b | Individual RCT (with narrow Confidence Interval”¡) | Individual inception cohort study with > 80% follow-up; CDR”  validated in a single population | Validating** cohort study with good” ” ”  reference standards; or CDR”  tested within one clinical centre | Prospective cohort study with good follow-up**** | Analysis based on clinically sensible costs or alternatives; systematic review(s) of the evidence; and including multi-way sensitivity analyses |
| 1c | All or none§ | All or none case-series | Absolute SpPins and SnNouts” “ | All or none case-series | Absolute better-value or worse-value analyses ” ” ” “ |
| 2a | SR (with homogeneity*) of cohort studies | SR (with homogeneity*) of either retrospective cohort studies or untreated control groups in RCTs | SR (with homogeneity*) of Level >2 diagnostic studies | SR (with homogeneity*) of 2b and better studies | SR (with homogeneity*) of Level >2 economic studies |
| 2b | Individual cohort study (including low quality RCT; e.g., <80% follow-up) | Retrospective cohort study or follow-up of untreated control patients in an RCT; Derivation of CDR”  or validated on split-sample§§§ only | Exploratory** cohort study with good” ” ”  reference standards; CDR”  after derivation, or validated only on split-sample§§§ or databases | Retrospective cohort study, or poor follow-up | Analysis based on clinically sensible costs or alternatives; limited review(s) of the evidence, or single studies; and including multi-way sensitivity analyses |
| 2c | “Outcomes” Research; Ecological studies | “Outcomes” Research |  | Ecological studies | Audit or outcomes research |
| 3a | SR (with homogeneity*) of case-control studies |  | SR (with homogeneity*) of 3b and better studies | SR (with homogeneity*) of 3b and better studies | SR (with homogeneity*) of 3b and better studies |
| 3b | Individual Case-Control Study |  | Non-consecutive study; or without consistently applied reference standards | Non-consecutive cohort study, or very limited population | Analysis based on limited alternatives or costs, poor quality estimates of data, but including sensitivity analyses incorporating clinically sensible variations. |
| 4 | Case-series (and poor quality cohort and case-control studies§§) | Case-series (and poor quality prognostic cohort studies***) | Case-control study, poor or non-independent reference standard | Case-series or superseded reference standards | Analysis with no sensitivity analysis |
| 5 | Expert opinion without explicit critical appraisal, or based on physiology, bench research or “first principles” | Expert opinion without explicit critical appraisal, or based on physiology, bench research or “first principles” | Expert opinion without explicit critical appraisal, or based on physiology, bench research or “first principles” | Expert opinion without explicit critical appraisal, or based on physiology, bench research or “first principles” | Expert opinion without explicit critical appraisal, or based on economic theory or “first principles” |

**Appendix 3.** quality evaluation of included studies case series studies:

| Numbers of studies | | 1 | 2 | 3 | 4 | 5 | 6 | 7 | 8 | 9 | 10 | 11 | 12 | 13 |
| --- | --- | --- | --- | --- | --- | --- | --- | --- | --- | --- | --- | --- | --- | --- |
| Study objective | 1. Was the hypothesis/aim/objective of the study clearly stated? | Y | Y | Y | Y | Y/N | Y | Y | Y | Y | Y | Y | Y | Y |
| Study design | 1. Was the study conducted prospectively? | Y | N | N | N | Y | N | N | N | ? | Y | ? | ? | N |
|  | 1. Were the cases collected in more than one centre? | N | N | N | ? | ？ | ? | N | N | N | ? | N | ? | N |
|  | 1. Were patients recruited consecutively? | ? | ? | ? | ? | ？ | ? | ? | ? | ? | ? | ? | ? | ? |
| Study population | 1. Were the characteristics of the patients included in the study described? | Y | Y | Y | Y | Y | Y | Y | Y | Y | Y | Y | Y | Y |
|  | 1. Were the eligibility criteria (i.e. inclusion and exclusion criteria) for entry into the study clearly stated? | Y | N | Y | Y/N | Y/N | Y/N | Y | N | Y/N | N | N | Y/N | N |
|  | 1. Did patients enter the study at a similar point in the disease? | Y | N | Y | N | N | N | N | N | Y | Y | Y | N | Y |
| Intervention and co-intervention | 1. Was the intervention of interest clearly described? | Y | Y | Y | Y/N | Y | Y | Y/N | Y | Y | Y | Y | N | Y/N |
|  | 1. Were additional interventions (co-interventions) clearly described? | Y | Y | Y | Y | Y | Y | Y | Y | Y | Y | Y | N | Y |
| Outcome measure | 1. Were relevant outcome measures established a priori? | Y | Y | Y | Y | Y | Y | Y | Y | Y/N | Y | Y | Y | Y/N |
|  | 1. Were outcome assessors blinded to the intervention that patients received? | ? | ? | ? | ? | ? | Y | ? | ? | ? | ? | ? | ? | ? |
|  | 1. Were the relevant outcomes measured using appropriate objective/subjective methods? | Y | Y | Y | Y | Y | Y | Y | Y | Y | Y | Y | Y | Y |
|  | 1. Were the relevant outcome measures made before and after the intervention? | Y | Y | Y | Y | Y | Y | Y | Y/N | Y | Y | Y | Y | Y |
| Statistical analysis | 1. Were the statistical tests used to assess the relevant outcomes appropriate? | Y | ? | Y | ? | ? | Y | Y | ? | Y | ? | ? | ? | ? |
| Results and conclusions | 1. Was follow-up long enough for important events to occur? | Y | Y | Y | Y | Y | Y | Y | Y | Y | Y | Y | Y | Y |
|  | 1. Were losses to follow-up reported? | Y | Y | Y | Y | Y | Y | Y | Y | Y | Y | Y | Y | Y |
|  | 1. Did the study provide estimates of random variability in the data analysis of the relevant outcomes? | Y | Y | Y | Y | Y/N | Y | Y/N | N | Y | N | Y/N | Y/N | Y |
|  | 1. Were the adverse events reported? | N | Y | N | Y | Y | Y | Y | Y | Y | N | N | N | Y |
|  | 1. Were the conclusions of the study supported by results? | Y | Y | Y | Y | Y | Y | Y | Y | Y | Y | Y | Y | Y |
| Competing interests and sources of support | 1. Were both competing interests and sources of support for the study reported? | N | Y | Y/N | Y | N | Y/N | N | N | N | N | N | N | N |
| total amount of “NO” | | 5 | 7 | 5 | 5 | 5 | 4 | 6 | 9 | 5 | 8 | 8 | 10 | 7 |
| Estimated risk of bias* | | M | H | M | M | M | M | H | VH | M | H | H | VH | H |

Y, Yes; Y/N Partial; N, No;. ?, Unclear; NA, not applicable; L, low risk of bias; M, moderate risk; H, high risk; VH, very high risk.

*: A study with 0–2 ‘no’ responses was considered to have a low risk of bias, 3–5 ‘no’ responses a moderate risk, 6–8 a high risk and ≥9 a very high risk of bias.

Numbers of studies：

1. 2016-Chen-Sartorius muscle-pedicle bone graft for osteonecrosis of the femoral head
2. 2014-Elmali-Vascular pedicled iliac bone grafting is effective in patients with an early stage of femoral head avascular necrosis
3. 2009-Chen-Vascularized iliac bone-grafting for osteonecrosis with segmental collapse of the femoral head
4. 2009-Babhulkar-Osteonecrosis of femoral head_ Treatment by core decompression and vascular pedicle grafting
5. 2009-Baksi-Long-term results of decompression and muscle-pedicle bone grafting for osteonecrosis of the femoral head
6. 2006-Zhao-Iliac graft vascularization for femoral head osteonecrosis
7. 2004-Nagoya-Predictive factors for vascularized iliac bone graft for nontraumatic osteonecrosis of the femoral head
8. 2001-Eisenschenk-Treatment of femoral head necrosis with vascularized iliac crest transplants
9. 1997-Hasegawa-Vascularized pedicle bone-grafting for nontraumatic avascular necrosis of the femoral head. A 5- to 11-year follow-up
10. 1997-Ishizaka-Vascularized iliac bone graft for avascular necrosis of the femoral head
11. 1996-Leung-Femoral head reconstruction and revascularization. Treatment for ischemic necrosis.
12. 1996-Wassenaar-Avascular osteonecrosis of the femoral head treated with a vascularized iliac bone graft
13. 1993-Iwata-Indications and results of vascularized pedicle iliac bone graft in avascular necrosis of the femoral head

Randomized controlled trial:

| 2016-Zhao-Vascularized bone grafting fixed by biodegradable magnesium screw for treating osteonecrosis of the femoral head | | |
| --- | --- | --- |
| Random sequence generation (selection bias) | Low risk | Every ONFH patient who underwent this operation was encoded with 1 or 2, “1” stands for the Mg group and “2” stands for the control group |
| Allocation concealment (selection bias) | Unclear risk | Not mentioned |
| Blinding of participants and personnel (performance bias) | High risk | There exists difficulty in blinding of participants and personnel for this operation belongs to invasive treatment |
| Blinding of outcome assessment (detection bias) | Low risk | One knowledgeable orthopedic surgeon conducted all quantitative measurements for statistical analysis |
| Incomplete outcome data (attrition bias) | Low risk | The entire follow up data were collected |
| Selective reporting (reporting bias) | Low risk | The entire result data were reported |
| Other bias | Low risk | We found no other risk of bias |
